# Supplementary material for: Zebrafish (Danio rerio) Prefer Undisturbed Shoals over Shoals Exposed to the Synthetic Alarm Substance Hypoxanthine-3N-oxide (C5H4N4O2)
Source: Biology (Basel). 2025 Feb 25;14(3):233. doi: 10.3390/biology14030233 (PMC11939818; doi:10.3390/biology14030233)
Supplement: Supplementary file 1 [file biology-14-00233-s001.zip › Supplemental File - S2 - Model Performance Table.pdf]

Supplemental File – S1

Table of Model Performance

Information criteria for model selection						
	3.2 Percent session time in zones	3.3a Percent of session time in motion	3.3b Percent of time in zone in motion	3.4a Percent of session time in freezing)	3.4b Percent of time in zone in freezing	3.5 Velocity during movement
**-2 Restricted Log Likelihood**	480.789	464.773	424.614	273.931	414.206	319.268
**Akaike's Information Criterion (AIC)**	498.789	482.773	442.614	291.931	432.206	337.268
**Hurvich and Tsai's Criterion (AICC)**	502.539	486.523	446.364	295.681	435.956	341.018
**Bozdogan's Criterion (CAIC)**	526.333	510.317	470.158	319.475	459.750	364.812
**Schwarz's Bayesian Criterion (BIC)**	517.333	501.317	461.158	310.475	450.750	355.812

According to SPSS, "the information criteria are displayed in smaller-is-better form."
